# Dietary Inclusion of an Anti-Mycotoxin Additive for Breeder Hens and Roosters Alleviates the Toxic Effects of Zearalenone on Fertility Rates and Chick Quality

**DOI:** 10.3390/toxins18070305

**Published:** 2026-07-14

**Authors:** Vinícius Moura, Mário Lopes, Lucas Butturi, Barbara Doupovec, Giovana Longhini, Marcelo Viviani, Fabricia Roque, Vitor Pais, Lúcio Araújo, Cristiane Araujo

**Affiliations:** 1 School of Veterinary Medicine and Animal Science, University of São Paulo, Pirassununga 13635-900, Brazil; viniciusmoura1996@hotmail.com; 2 School of Animal Science and Food Engineering, University of São Paulo, Pirassununga 13635-900, Brazil; mhsl.br@usp.br (M.L.); lucasbutturi@gmail.com (L.B.); giovanamachado@tamu.edu (G.L.); vivianimarcelo2001@gmail.com (M.V.); jotapvargas@gmail.com (F.R.); vitorsouza@usp.br (V.P.); lfaraujo@usp.br (L.A.); 3 dsm-firmenich, Animal Nutrition and Health R&D Center, 3430 Tulln, Austria; barbara.doupovec@dsm-firmenich.com

**Keywords:** eggshell thickness, embryonic development, hatchability, offspring, sperm cell morphology

## Abstract

Mycotoxins are a worldwide hazard to animal feed and capable of deleterious effects on poultry. The present study aimed to evaluate the effects of an anti-mycotoxin feed additive on productive and reproductive parameters of breeder hens and roosters fed diets experimentally contaminated with zearalenone (ZEN). A total of 288 hens and 32 roosters (Cobb 500) were randomly distributed in one of four dietary treatments, with nine hens and a rooster per pen and eight pens per treatment: control (C); C + anti-mycotoxin (AM) additive; C + ZEN; and C + AM + ZEN. The trial lasted from 28 to 65 weeks of age. Egg production, egg quality, fertility, hatchability, and hatch rates of fertile eggs were evaluated, as were sperm morphology and abnormalities, and chick quality. ZEN inclusion did not affect egg production but had harmful effects on albumen, egg quality and weight, eggshell thickness, egg fertility and hatchability, and chick length and quality, compared to C, with this negative effect partially alleviated by AM inclusion. Both AM and ZEN, however, decreased spermatic concentration, without affecting sperm morphology or abnormality. The anti-mycotoxin additive effectively mitigated the deleterious effects of ZEN on reproductive parameters and egg quality indexes when fed to breeder hens.

## 1. Introduction

Increasing egg quality and, consequently, the number of viable chicks per hen has become a necessity in modern poultry production; it is vital to control factors that deeply affect hen physiological development and productivity. Among these factors, hen nutrition is fundamental for maintaining the bird’s health and, along with hen age, body weight, and stressful environmental factors, directly affects progeny quality [1]. The maternal diet is intrinsically correlated with the nutritional content of the yolk and, therefore, is also responsible for embryonic development and post-hatch growth [1,2,3].

Hence, rigorous control of feed quality and composition has become a major concern, as many forms of feed contamination can occur and interfere with bird performance and, consequently, progeny quality, with mycotoxin contamination being a main hazard in feed control [4]. Mycotoxins are secondary metabolites produced by fungi during their development [5] and can cause neurotoxicity, immunosuppression, teratogenicity, and deleterious reproductive effects when ingested through feed [6]. Among the different fungal metabolites, zearalenone (ZEN) is a mycotoxin produced by Fusarium species and is frequently found in grains such as oats, rice, barley, corn, and wheat [7,8]. The chemical structure of ZEN is similar to that of the natural hormone 17β-estradiol, allowing it to bind to estrogen receptors in target cells [9,10]. ZEN acts directly on the reproductive system of birds, promoting alterations such as irregular gametogenesis, impaired embryo development, reduced egg production, and hypertrophy of the reproductive tract, leading to abnormalities and reduced performance [11,12].

Previous studies have shown that ZEN contamination in feed can affect bird productivity and reproductive function. Allen and coworkers [13] reported decreased Haugh units, reduced bird fertility, and increased early embryonic mortality in eggs laid by hens fertilized by ZEN-fed roosters. Yegani and colleagues [14] also reported an increase in early embryonic mortality in eggs laid by hens fed ZEN-contaminated diets.

Given the adverse effects of ZEN and other mycotoxins on birds, research on interventions and solutions for toxin control has been conducted, including anti-mycotoxin feed additives [15]. Some of those additives include: (1) Volcanic-based clays, such as bentonite, which can bind to mycotoxin molecules in the intestinal lumen, forming clay-toxin complexes that are eliminated in the excreta and, therefore, preventing proper mycotoxin conversion and, therefore, biological effects [16]. (2) Live probiotic bacteria, such as *Roseburia* spp., *Eubacterium* spp., *Bifidobacterium* spp., and yeast species, such as *Trichosporon mycotoxivorans*, which can biodegrade and hydrolyze some mycotoxin compounds, resulting in less toxic or non-toxic secondary metabolites in monogastrics [17,18,19,20]. And (3) Direct-fed enzymes, which can also degrade the toxic substances directly in the intestinal lumen. Although quite common in swine and broiler chickens, the effects of anti-mycotoxin additives for breeder hens and roosters and their implications are not yet fully understood. Therefore, this study aimed to evaluate the effects of a feed additive containing bentonite clay, *Eubacterium* spp., zearalenone hydrolase, and fumonisin esterase on breeder hens and roosters challenged with ZEN, including the impact on bird productivity, reproductive performance, and progeny quality throughout the birds’ life cycles.

## 2. Results

The results for egg production during the entire trial period are shown in Table 1. There was no effect of ZEN contamination or AM use (*p* > 0.05) on total egg production.

Regarding egg quality analysis, differences were observed across all three evaluated periods, as shown in Table 2. At 33 and 44 weeks of age, the highest albumen and Haugh unit values were observed for eggs laid by hens fed the C + AM + ZEN diets (*p* < 0.05), with those eggs also showing the thickest eggshell at 44 weeks of age. At 33 weeks, the C group showed the lowest albumen height and Haugh unit values among all groups, but at 44 weeks, the lowest values for these parameters were observed in the C + ZEN group. At 66 weeks of age, the C + AM group produced the heaviest eggs and thickest eggshells between treatments, followed by C, C + AM + ZEN, and C + ZEN (*p* < 0.05).

As for reproductive indexes, no differences were observed between treatments for any of the evaluated parameters at 37 weeks of age (*p* > 0.05, Table 3). However, at both 47 and 62 weeks of age, ZEN contamination severely reduced egg fertility, an effect that was alleviated by the inclusion of AM in the ZEN-contaminated diet. ZEN contamination also decreased hatchability rate at 47 weeks, while the C group showed the highest pipped egg rate between treatments at 62 weeks of age (*p* < 0.05). No differences in embryonic mortality were observed amongst treatments in all evaluated periods (*p* > 0.05).

In all evaluated periods, differences were observed between treatments regarding chick length (Table 4). In hatched eggs from the 40-week-old hens, ZEN contamination negatively affected the newly hatched chick length (*p* < 0.001), regardless of AM supplementation. In hatched eggs from 50- and 65-week-old hens, the longest chicks were hatched from hens fed ZEN-contaminated diets with inclusion of AM, while the shortest chicks were the ones hatched from hens fed the ZEN-contaminated diet only (*p* < 0.01). Open or unhealed navels were also different in later hatched eggs, being the highest ratios observed for C + ZEN chicks at the second hatch, and for C chicks at the third hatch (*p* < 0.05).

The results concerning semen quality and sperm morphology are shown in Table 5. In the first analysis at 30 weeks of age, roosters fed diets containing AM showed the lowest sperm concentration (*p* < 0.05) but also the highest motility percentage (*p* < 0.001). No differences were observed amongst treatments for any of the evaluated parameters in the second analysis, at 45 weeks of age. However, at 63 weeks of age, roosters fed the C diet showed the highest subjective motility, followed by C + AM, C + ZEN, and C + AM + ZEN (*p* < 0.05).

Results concerning sperm cell morphological analysis are shown in Table 6. No difference in treatment effects was observed across any of the evaluated bird ages (*p* > 0.05).

**Figure 1:**
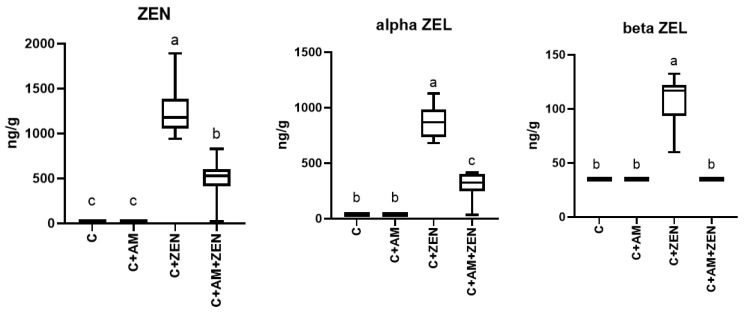
Concentration of ZEN and its main metabolites in breeder hens’ excreta. ^1^ Control (C)—standard corn and soybean-based diet; C + AM—control diet with the inclusion of anti-mycotoxin additive; C + ZEN—control diet experimentally contaminated with zearalenone; C + AM + ZEN—control diet experimentally contaminated with zearalenone and with inclusion of anti-mycotoxin feed additive. ^2^ Means with different superscripts within the same row significantly differ according to the Tukey test (*p* < 0.05).

**Figure 2:**
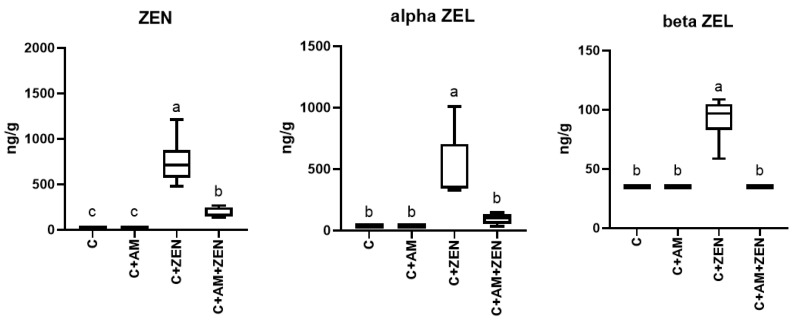
Concentration of ZEN and its main metabolites in roosters’ excreta. ^1^ Control (C)—standard corn and soybean-based diet; C + AM—control diet with the inclusion of anti-mycotoxin additive; C + ZEN—control diet experimentally contaminated with zearalenone; C + AM + ZEN—control diet experimentally contaminated with zearalenone and with inclusion of anti-mycotoxin feed additive. ^2^ Means with different superscripts within the same row significantly differ according to the Tukey test (*p* < 0.05).

The measured concentrations for ZEN and its main metabolites, alpha and beta ZEL, in hens’ and roosters’ excreta are shown in Figure 1 and Figure 2, respectively. The AM additive was able to reduce ZEN and ZEL excretion in the feces by more than half when compared to the concentrations found in the excreta from C + ZEN birds. The concentration of ZEN and ZEL in the rooster excreta was also lower than the measured values for hen excreta.

## 3. Discussion

The present study aimed to assess the effects of a dietary anti-mycotoxin additive on the productive and reproductive performance of experimentally ZEN-exposed broiler breeder hens and roosters. No significant changes were observed in total egg production between treatments in the present study (Table 1), similar to the results observed in laying hens by [12,21], but disagreeing with [22,23,24] who reported significant decreases in laying performance when laying hens were fed diets containing ZEN. It is possible that higher ZEN doses in the latter studies, along with differences in breed, feed regime, and metabolism between layers and broiler breeders, could explain the discrepancies in the literature regarding egg production. Since laying hens usually have access to unrestricted feed, cumulative ZEN intake could be more expressive than in breeders, which were fed once a day in highly restrictive quantities.

Concerning external and internal egg quality assays, interestingly, eggs from C + AM + ZEN group showed the highest values for albumen height and consequently Haugh unit at the first and second evaluations, at 33 and 44 weeks of bird age, while the lowest values for those measurements were observed for C + ZEN at 44 weeks of age, similarly to the results found by [12,25], whom reported decreases in albumen height and Haugh unit in laying hens fed different mycotoxins. Hence, the negative effects of ZEN contamination on internal egg quality were likely mitigated by the inclusion of the anti-mycotoxin additive, as observed in Table 2. Despite the known effects of mycotoxins, such as aflatoxin, on egg quality [25], few studies report the interference of ZEN on internal egg quality. Since ZEN can bind estrogen receptors and thus compete with estradiol, the estrogen-signaling pathways are compromised during ZEN contamination [12], leading to alterations in albumen formation. Since estradiol induces the expression of major egg white proteins [26,27], ZEN may contribute to reducing total albumen height, and therefore interfere with Haugh unit values, as well as egg weight, since the albumen can account for up to 60% of total egg weight [28].

As for the analysis at 66 weeks of age, ZEN significantly reduced both eggshell thickness (*p* < 0.001) and egg weight (*p* < 0.05). It is expected that, as hen age increases, a concomitant increase in egg weight occurs, since there is higher deposition of hepatic-synthesized substances in a smaller number of viable ovarian follicles [29]. ZEN, as well as other mycotoxins, has been reported to promote hepatic dysfunction and impairment, including reductions in lipid and cholesterol synthesis [30], resulting in lower yolk deposition and lower egg weight [31]. By reducing egg size and weight, an increase in eggshell thickness is expected to occur, since there is a proportional reduction in the internal content of the egg without interfering with eggshell calcium deposition [32]. However, in the present study, hens fed with C + ZEN showed reduced eggshell thickness at both 44 and 66 weeks of age. Authors hypothesize that this decrease is due to impaired liver function in metabolizing core nutrients for shell quality, such as vitamin D, calcium, and zinc, caused by ZEN’s high toxicity [33,34]. In the present study, dietary inclusion of AM alleviated the toxic effects of ZEN on internal egg quality, egg weight, and eggshell thickness. This was likely due to the properties of the compounds found in AM that cannot only bind with ZEN in the intestinal lumen, allowing for toxin excretion and thus reducing the concentrations available for absorption [19,35], but also by directly detoxifying ZEN by metabolizing its molecule into non-toxic metabolites [36], being these effects able to alleviate the oxidative stress caused by ZEN on liver and kidneys [31,37]. It is important to note that the main detoxification pathways in chickens involve reduction and sulfation [38]. The inclusion of ZEN hydrolase in the AM additive can provide additional metabolic pathways, thereby alleviating liver oxidative stress during detoxification [39].

Dietary ZEN contamination negatively affected both hatch indexes and chick quality parameters in all three hatches, as shown in Table 3 and Table 4. For the first hatch, despite no differences in fertility, hatchability, or fertile hatch rate, chicks from C + ZEN and C + AM + ZEN-fed hens had the shortest lengths among treatment groups (*p* < 0.001). Chick length is an important chick quality measurement, as it correlates with post-hatch performance and meat yield [40], suggesting that chick length assessment can be an early indicator of broiler chicken performance. Authors hypothesize that, because ZEN can impair liver function and nutrient deposition in the yolk, chicks have fewer nutrient resources for embryonic development, leading to smaller chicks at hatch.

As for the second hatch, ZEN decreased both fertility and egg hatchability rates, an effect that was alleviated by AM inclusion in the ZEN-contaminated diets. Due to hormonal balance disruptions promoted by ZEN binding with estrogen receptors, impairments in granulosa cell development, as well as increases in oxidative stress and granulosa cell apoptosis can occur [41,42], which may contribute to the lower egg fertility observed in the ZEN-challenged hens. Despite not directly investigating how AM was able to alleviate the observed ZEN effects on fertility and hatchability, the authors hypothesize that, since compounds in the additive, such as ZEN hydrolase and bentonite clay, reduce the overall ZEN concentration in the hen organism, allowing for an indirect preservation of reproductive function, a lower concentration of ZEN is competing for estrogen receptors. Chick length was also decreased, and open navel percentage was increased in chicks hatched from C + ZEN diets. Compared with C + ZEN, C + AM + ZEN attenuated the harmful effects of mycotoxin contamination (*p* < 0.001). In the third hatch, ZEN exposure severely compromised fertility and altered chick length, effects that were further enhanced by dietary AM (*p* < 0.05). Reference [43] reported no differences in chick body weight at hatch between hens fed control diets and those fed mycotoxin-contaminated diets. However, the progeny from hens fed the contaminated diets showed significantly lower body weight at 14 d than chicks from control hens, indicating that maternal dietary mycotoxin contamination can promote harmful effects on the offspring even weeks after hatch. In the present study, despite the newly hatched chicks not being raised after hatch, the effects of ZEN-exposure in maternal diets showed deleterious effects to day-old chicks. Similarly to our findings, Yuan and coworkers [44] reported a decrease in hatchability rates, as well as lower newly hatched chick weight in eggs experimentally exposed to ZEN.

The effects of ZEN and AM on male broiler breeder reproductive performance were also evaluated and are shown in Table 5 and Table 6. No differences in sperm morphology and abnormality percentages were observed between treatment groups at any of the evaluated time points (*p* > 0.05). This was not expected by the authors, since most mycotoxins are known to impair testicular function, suppress sperm production, and promote abnormality in sperm cells [45]. The observed result is likely due to the lower ZEN dosage fed to roosters, which were also under severe feed restriction. All treatment groups also showed lower sperm concentration than the control group in the first assay, with higher subjective motility, which is an expected reaction to lower concentrations, as the decrease in concentration also decreases oxidative stress, leading to an increase in motility [46]. In the third sperm quality assay, all treatment groups showed lower subjective motility percentages than the control group, with the lowest observed in the C + AM + ZEN group (*p* < 0.05). To our knowledge, few studies on the specific effects of ZEN on rooster fertility are available in the literature; the present study is the first to evaluate the effects of experimental ZEN contamination on rooster semen quality and morphology. Reference [13] evaluated different dietary concentrations of ZEN for male New Hampshire roosters, and no effects on fertility and hatch of fertile eggs were observed.

The inclusion of AM in ZEN-contaminated diets partially reduced the final concentrations of ZEN and ZEL in excreta. The enzymatic compound ZEN hydrolase present in AM is capable of degrading and detoxifying ZEN in the intestinal lumen, allowing ZEN and ZEL to be converted into non-toxic alternative metabolites [47,48]. Bentonite, present in the AM additive, can also bind to mycotoxins, forming bentonite-toxin complexes that are excreted in fecal material, mitigating the potential harmful effects of mycotoxins on the intestinal epithelium [49]. Overall, the decreased concentrations of ZEN and ZEL in the excreta of both hens and roosters when AM was added to ZEN-contaminated diets indicate the efficacy of AM in degrading and neutralizing some of the harmful metabolites converted during ZEN hepatic metabolism.

The present study also faced limitations, such as co-contamination with fumonisin in all experimental diets, which could potentially interfere with the observed results for some egg quality parameters, as well as the hatchability and male fertility parameters observed mainly in late-age birds. The lack of different dosages for ZEN and AM inclusion can also limit the ability to observe dose–response relationships in key parameters of bird physiology. In addition, the trial lasted from 28 to 65 weeks of age, but mycotoxin testing was conducted only at 40 weeks, potentially missing important co-contaminations in the basal diets.

## 4. Conclusions

Broiler breeder nutrition expresses direct influence on progeny quality and development. Under the conditions of this trial, the inclusion of a dietary anti-mycotoxin additive attenuated the harmful effects of zearalenone on productive and reproductive performance parameters. These benefits were observed across key areas, including internal and external egg quality, fertility, hatchability, and post-hatch chick quality. Collectively, these factors contribute to adequate egg fertility throughout the breeder hen’s life cycle and are key to progeny quality, highlighting the importance of proper controls and solutions for mycotoxin challenges. Similarly, male breeder nutrition plays a key role in reproductive success. While ZEN contamination was shown to affect sperm quality, the inclusion of the product in this study highlights the importance of continued innovation and optimization to further support reproductive performance under challenging conditions of fungal contamination in feed ingredients.

## 5. Materials and Methods

The study was conducted at the Poultry Science Laboratory of the School of Veterinary Medicine and Animal Science of the University of São Paulo, Pirassununga, Brazil. The study protocol met the guidelines approved by the institutional animal care and use committee (protocol number 9838181120).

A total of 288 Cobb broiler breeder hens and 32 roosters were acquired from a commercial farm at 22 weeks of age, and after a six-week adaptation period, they received experimental diets from 28 up to 65 weeks of age. Birds were randomly distributed in four dietary treatments with eight replicates each, with each pen containing nine hens and a rooster. Dietary treatments were control (C)—standard corn and soybean-based diet; C + anti-mycotoxin additive (AM, 2 kg/MT); C + ZEN (1000 ppb for hens and 500 ppb for roosters); and C + AM (2 kg/MT) + ZEN (1000 ppb for hens and 500 ppb for roosters). Management, lightning, and feeding practices were followed according to the lineage manual. Before the start of the trial, all birds were individually weighed to ensure body weight homogeneity between treatments, with additional weight measurements at 45 and 64 weeks. Diets were isonutritious, isocaloric, and corn-, soybean-, and wheat-based, formulated according to the nutritional exigencies proposed by [50], as seen in Table 7. Feed was provided once daily, with amounts adjusted according to bird age, as recommended by the lineage guidelines.

The anti-mycotoxin additive used in the experimental diets was Mycofix Plus^®^ 5.Z (Herzogenburg, Austria), containing bentonite, *Eubacterium* spp., fumonisin esterase, and a zearalenone hydrolase. Zearalenone was incorporated in feed by including fungal culture material (BiMM—Bioactive Microbial Metabolites Group) containing 1.122 ZEN/kg. Since male birds appear to be more susceptible to toxic reproductive effects from mycotoxins [13,51], half of the inclusion rate of hens was used for roosters. The trial was carried out in an experimental barn with 32 pens, with dimensions of 2.4 × 1.6 × 2.1 m, equipped with fans, through feeder adaptations to prevent the rooster from accessing hen feed, nipple drinkers, and a tall feeder only accessed by the rooster, as well as 3 nest boxes and wood shavings as bedding.

All experimental diets were tested for ten major mycotoxins in poultry feedstuffs in Brazil, including ZEN. Testing was conducted at 40 weeks of age. Feed mycotoxin analysis is presented in Table 8.

Bird performance and egg quality

Productive performance was evaluated based on total egg production from 28 to 65 weeks of age, expressed as a percentage. Daily egg production was registered, and egg/hen/day production was measured by dividing the total number of eggs laid daily by the number of hens in each replicate, multiplied by 100.

Egg quality parameters were evaluated at 33, 44, and 63 weeks of age by collecting all eggs on each evaluation day. Internal and external egg quality characteristics were analyzed using a Digital Egg Tester (DET-6000, NABEL Co., Ltd., Kyoto, Japan), including egg weight (g), albumen height (mm), Haugh unit, egg yolk color, eggshell breaking strength resistance (kgf), and eggshell thickness (mm).

Incubation and bird reproductive performance

During weeks 37, 47, and 62 of age, all clean, non-cracked eggs were collected for seven days, properly identified by treatment and replicate number, and placed in a 16 °C room until situated in the incubator, following standard practices. After 21 days of incubation, all newly hatched chicks were counted and registered, and 40 chicks of each sex were randomly selected to evaluate length (cm), presence of open, unhealed navels, dehydration signs, or red hocks incidence. Unhatched eggs were cracked to determine embryonic mortality, classified as early (1–7 d), mid (8–14 d), late (15–21 d), infertile, contaminated, or pipped-but-unhatched eggs with dead embryos.

Breeder hen reproductive performance was assessed by egg fertility rate (number of fertile eggs divided by total eggs incubated, multiplied by 100), hatchability rate (number of eggs hatched divided by total eggs incubated, multiplied by 100), hatch fertility rate (number of eggs hatched divided by total fertile eggs, multiplied by 100), newly hatched chick quality measurements, and embryo mortality analysis.

Sperm analysis and semen physical characteristics

At 30, 45, and 63 weeks of age, roosters were placed in separate pens for a total of 3 days to completely avoid coupling and allow for fresh semen collection. Each rooster was individually stimulated by abdominal massage, and semen was collected in conical, graduated tubes to avoid contamination via excreta or feathers. Tubes were kept at 30 °C to avoid alterations in morphology, and seminal volume was determined directly by the amount of semen read by the graduations on the semen tube, expressed in mL. Immediately after collection, 4 μL of fresh semen was placed between a slide and coverslip for evaluation of motility and spermatic vigor under optical microscopy (Nikon E200, Tokyo, Japan) at 100× magnification. Motility was expressed by the percentage of motile sperm cells in a sample, and vigor was evaluated based on curvilinear velocity, ranked on a scale from 0 to 5, with 0 being the complete absence of movement and 5 a vigorous, progressive, and uniform movement, as described by Celeghini and colleagues [52].

For evaluation of sperm morphology and concentration, another sample was diluted in a buffered saline formaldehyde solution (4% formaldehyde in DPBS). The humid chamber technique was used, in which a droplet (4 μL) of diluted semen was loaded into a slide chamber and observed by differential interference contrast microscopy (DIC, Nikon 80i, Tokyo, Japan) at 1000× magnification, with 200 sperm cells per sample evaluated. Abnormalities were then classified into defects in the acrosome, head, middle piece, tail, and other categories, such as cytoplasmic protrusions, isolated heads, and teratological defects. Sperm analyses were conducted using the computer-assisted sperm analysis system (CASA) model IVOS II (Version MK5, Hamilton Thorne, Beverly, MA, USA) and the Animal Breeder II software (Version 1.11.0, Hamilton Thorne, USA), with configurations properly set for rooster sperm.

ZEN and metabolites excreta concentration

At 64 weeks of age, one hen and one rooster per pen were allotted in individual metabolic cages with removable metal trays for excreta collection. All excreta were collected from each cage on a single day, and ZEN, alpha-zearalenol (ZEL), and beta-ZEL concentrations (ng/g) from the total excreta content were evaluated according to the methodology described in [53].

Statistical analysis

All data obtained in the trial were analyzed using SAS (Statistical Analysis System, version 9.4; SAS Institute Inc., Cary, NC, USA). Data were submitted to ANOVA with a significance level of 0.05. When significant, LSMEANS were separated between treatments by the post hoc Tukey test.

## Figures and Tables

**Table 1:** Total egg production period of dietary ZEN-challenged broiler breeder hens with or without a dietary anti-mycotoxin feed additive.

	Treatments				SEM ^2^	*p*
	Control ^1^	C + AM	C + ZEN	C + AM + ZEN		
Total egg production (%) 28–65 weeks	69.04	66.20	65.75	66.55	0.93	0.620

^1^ Control (C)—standard corn and soybean-based diet; C + AM—control diet with the inclusion of anti-mycotoxin additive; C + ZEN—Control diet experimentally contaminated with zearalenone; C + AM + ZEN—control diet experimentally contaminated with zearalenone and with inclusion of anti-mycotoxin feed additive. ^2^ Standard error of the mean.

**Table 2:** Internal and external egg quality of dietary ZEN-challenged broiler breeder hens with or without a dietary anti-mycotoxin feed additive at 33, 44, and 63 weeks of age.

	Treatments				SEM ^2^	*p* ^3^
	Control ^1^	C + AM	C + ZEN	C + AM + ZEN		
33 Weeks						
Egg weight (g)	63.68	64.56	63.49	64.00	0.24	0.409
Albumen height (mm)	7.52 ^d^	7.96 ^c^	8.82 ^b^	9.25 ^a^	0.17	0.001
Haugh unit	88.26 ^c^	91.16 ^b^	95.06 ^a^	94.90 ^a^	0.93	0.026
Eggshell resistance (kgf)	3.84	4.14	4.06	4.07	0.04	0.090
Eggshell thickness (mm)	0.376	0.384	0.376	0.381	0.002	0.346
44 Weeks						
Egg weight (g)	70.03	71.27	71.93	70.69	0.30	0.141
Albumen height (mm)	8.07 ^c^	8.65 ^b^	7.75 ^d^	10.73 ^a^	0.024	<0.001
Haugh unit	86.70 ^b^	87.72 ^b^	83.83 ^c^	100.07 ^a^	1.15	<0.001
Eggshell resistance (kgf)	4.01	4.17	4.26	4.23	0.06	0.506
Eggshell thickness (mm)	0.395 ^b^	0.371 ^d^	0.389 ^c^	0.402 ^a^	0.002	<0.001
63 Weeks						
Egg weight (g)	74.52 ^b^	75.02 ^a^	71.95 ^d^	74.01 ^c^	0.42	0.045
Albumen height (mm)	7.56	7.65	7.43	7.52	1.17	0.915
Haugh unit	82.22	83.02	82.09	82.56	0.50	0.976
Eggshell resistance (kgf)	3.63	3.55	3.56	3.56	0.87	0.983
Eggshell thickness (mm)	0.396 ^b^	0.398 ^a^	0.376 ^d^	0.381 ^c^	0.01	<0.001

^1^ Control (C)—standard corn and soybean-based diet; C + AM—control diet with the inclusion of anti-mycotoxin additive; C + ZEN—control diet experimentally contaminated with zearalenone; C + AM + ZEN—control diet experimentally contaminated with zearalenone and with inclusion of anti-mycotoxin feed additive. ^2^ Standard error of the mean. ^3^ Means with different superscripts within the same row significantly differ according to the Tukey test (*p* < 0.05).

**Table 3:** Hatch of fertile eggs, hatchability, fertility, and embryonic mortality rates of dietary ZEN-challenged broiler breeder hens with or without a dietary anti-mycotoxin feed additive.

	Treatments				SEM ^2^	*p* ^3^
	Control ^1^	C + AM	C + ZEN	C + AM + ZEN		
37 Weeks						
Hatch of fertile, %	94.30	93.19	92.25	93.95	1.72	0.79
Hatchability, %	88.98	87.09	77.22	91.52	4.89	0.18
Fertility, %	94.12	92.67	83.53	97.33	4.26	0.14
Early mortality, %	1.12	3.11	2.82	3.83	0.93	0.18
Mid mortality, %	0.62	0.00	0.00	0.27	0.29	0.48
Late mortality, %	2.16	2.15	1.98	0.89	0.88	0.72
Pipped, %	1.74	0.32	1.42	0.83	0.71	0.52
*N* of hatched	188	249	269	289		
47 Weeks						
Hatch of fertile, %	89.67	83.52	80.21	93.49	4.41	0.19
Hatchability, %	82.67 ^a^	68.64 ^a^	55.06 ^b^	87.08 ^a^	7.15	0.02
Fertility, %	93.86 ^a^	78.90 ^ab^	67.51 ^b^	93.05 ^a^	6.19	0.03
Early mortality, %	6.26	1.67	9.16	4.86	2.26	0.21
Mid mortality, %	0.00	0.00	0.96	0.00	0.32	0.20
Late mortality, %	2.00	2.36	1.70	0.46	1.01	0.55
Pipped, %	1.86	1.03	0.87	0.66	1.00	0.80
*N* of hatched	150	150	150	150		
62 Weeks						
Hatch of fertile, %	81.01	83.49	74.10	90.25	7.33	0.52
Hatchability, %	75.62	75.32	53.34	82.24	7.32	0.08
Fertility, %	93.11 ^a^	89.73 ^ab^	67.11 ^b^	90.59 ^ab^	6.22	0.04
Early mortality, %	4.74	8.63	8.78	3.94	3.99	0.73
Mid mortality, %	0.00	0.00	0.00	0.00	0.00	1.00
Late mortality, %	3.44	4.15	2.72	2.72	1.73	0.89
Pipped, %	8.59 ^a^	1.29 ^b^	1.39 ^b^	0.70 ^b^	1.91	0.04
*N* of hatched	101	102	79	122		

^1^ Control (C)—standard corn and soybean-based diet; C + AM—control diet with the inclusion of anti-mycotoxin additive; C + ZEN—control diet experimentally contaminated with zearalenone; C + AM + ZEN—control diet experimentally contaminated with zearalenone and with inclusion of anti-mycotoxin feed additive. ^2^ Standard error of the mean. ^3^ Means with different superscripts within the same row significantly differ according to the Tukey test (*p* < 0.05).

**Table 4:** Newly hatched chick quality of dietary ZEN-challenged broiler breeder hens with or without a dietary anti-mycotoxin feed additive.

	Treatments				SEM ^2^	*p* ^3^
	Control ^1^	C + AM	C + ZEA	C + AM + ZEA		
40 Weeks						
Chick length (cm)	18.63 ^a^	18.61 ^a^	18.36 ^b^	18.04 ^c^	0.04	<0.001
Open navel, %	4.94	3.70	4.94	7.41	0.01	0.760
Dehydration,%	0.00	0.00	0.00	0.00	0.00	-
Red hocks, %	1.23	0.00	0.00	0.00	0.01	0.393
50 Weeks						
Chick length (cm)	18.94 ^b^	18.93 ^b^	18.78 ^c^	19.08 ^a^	0.03	0.009
Open navel, %	0.14 ^c^	0.08 ^b^	0.25 ^d^	0.02 ^a^	0.02	<0.001
Dehydration,%	0.00	0.02	0.02	0.00	0.01	0.573
Red hocks, %	0.00	0.02	0.02	0.00	0.01	0.573
65 Weeks						
Chick length (cm)	18.25 ^b^	18.76 ^a^	15.71 ^c^	18.84 ^a^	0.21	<0.001
Open navel, %	0.25 ^d^	0.12 ^b^	0.06 ^a^	0.17 ^c^	0.02	0.028
Dehydration,%	0.00	0.00	0.02	0.00	0.01	0.391
Red hocks, %	0.03	0.00	0.02	0.02	0.01	0.567

^1^ Control (C)—standard corn and soybean-based diet; C + AM—control diet with the inclusion of anti-mycotoxin additive; C + ZEN—control diet experimentally contaminated with zearalenone; C + AM + ZEN—control diet experimentally contaminated with zearalenone and with inclusion of anti-mycotoxin feed additive. ^2^ Standard error of the mean. ^3^ Means with different superscripts within the same row significantly differ according to the Tukey test (*p* < 0.05).

**Table 5:** Sperm quality and morphology of dietary ZEA-challenged broiler breeder roosters with or without a dietary anti-mycotoxin feed additive.

30 Weeks						
Parameters	C ^1^	C + AM	C + ZEN	C + AM + ZEN	SEM ^2^	*p* ^3^
Volume (mL)	0.22	0.20	0.15	0.19	0.09	0.664
Concentration (million per mL)	3016 ^a^	2005 ^bc^	2557 ^ab^	1334 ^c^	1005	0.024
Subjective attributes						
Vigor, %	3.63	3.88	3.60	3.83	0.26	0.154
Motility, %	71.67 ^c^	79.38 ^a^	76.00 ^b^	79.17 ^ab^	3.36	<0.001
Morphological attributes						
Bent tail, %	18.45	11.50	8.91	10.61	2.80	0.716
Coiled tail, %	0.88	0.76	0.00	0.00	0.26	0.551
Distal cytoplasmic droplet, %	8.77	2.87	6.22	8.61	1.56	0.440
Distal midpiece reflex, %	9.51	6.42	2.68	2.69	1.67	0.307
45 Weeks						
Volume (mL)	0.26	0.22	0.23	0.25	0.14	0.967
Concentration (million per mL)	4978	3875	2678	2993	1363	0.053
Subjective attributes						
Vigor, %	3.30	3.83	3.88	3.75	0.54	0.320
Motility, %	77.50	77.50	80.00	77.50	4.41	0.822
Morphological attributes						
Motility, %	80.92	89.09	89.60	92.26	2.74	0.07
Total sperm cells (million per mL)	4222.01	2895.01	2426.87	2956.39	753.79	0.49
Bent tail, %	0.38	0.54	0.80	0.18	0.29	0.57
Coiled tail, %	0.04	0.15	0.04	0.02	0.07	0.83
Distal cytoplasmic droplet, %	0.27	0.17	0.11	0.21	0.05	0.12
Distal midpiece reflex, %	0.43	0.20	0.13	0.10	0.10	0.19
63 Weeks						
Volume (mL)	0.17	0.08	0.10	0.10	0.6	0.193
Concentration (million per mL)	-	-	-	-	-	-
Subjective attributes						
Vigor, %	3.00	3.00	3.20	2.50	0.37	0.147
Motility, %	75.00 ^a^	74.00 ^b^	70.00 ^c^	55.00 ^d^	7.93	0.004
Morphological attributes						
Motility, %	84.02	88.66	88.96	88.71	5.34	0.89
Total sperm cells (million per mL)	3350.19	1920.66	1027.92	713.68	706.93	0.14
Bent tail, %	0.53	0.15	0.76	0.33	0.37	0.59
Coiled tail, %	0.10	0.03	0.10	0.13	0.07	0.74
Distal cytoplasmic droplet, %	0.14	0.16	0.16	0.15	0.06	0.95
Distal midpiece reflex, %	0.23	0.08	0.11	0.12	0.04	0.10

^1^ Control (C)—standard corn and soybean-based diet; C + AM—control diet with the inclusion of anti-mycotoxin additive; C + ZEN—control diet experimentally contaminated with zearalenone; C + AM + ZEN—control diet experimentally contaminated with zearalenone and with inclusion of anti-mycotoxin feed additive. ^2^ Standard error of the mean. ^3^ Means with different superscripts within the same row significantly differ according to the Tukey test (*p* < 0.05).

**Table 6:** Morphological analysis of sperm cells from dietary ZEN-challenged broiler breeder roosters with or without a dietary anti-mycotoxin feed additive.

45 Weeks						
Variables	C ^1^	C + AM	C + ZEN	C + AM + ZEN	SEM ^2^	*p*
Acrosome	-	-	-	-	-	-
Head	3.797	2.519	4.123	1.412	0.882	0.126
Middle piece	3.390	1.151	0.944	0.751	0.923	0.226
Tail	1.916	0.809	0.873	1.293	0.608	0.481
Abnormalities	9.176	4.504	5.715	3.772	1.662	0.118
Normality	90.904	95.494	94.299	96.234	1.666	0.128
N of birds	7	7	4	7		
63 Weeks						
Variables	C	C + AM	C + ZEN	C + AM + ZEN	SEM	*p*
Acrosome	-	-	-	-	-	-
Head	15.588	21.396	22.042	16.328	2.912	0.125
Middle piece	1.344	0.750	0.750	0.790	0.626	0.871
Tail	0.132	0.199	0.172	0.121	0.137	0.460
Abnormalities	17.791	22.246	18.286	15.263	3.042	0.536
Normality	82.530	77.763	77.273	84.214	3.119	0.224
N of birds ^3^	4	5	5	2		

^1^ Control (C)—standard corn and soybean-based diet; C + AM—control diet with the inclusion of anti-mycotoxin additive; C + ZEA—control diet experimentally contaminated with zearalenone; C + AM + ZEA—control diet experimentally contaminated with zearalenone and with inclusion of anti-mycotoxin feed additive. ^2^ Standard error of the mean. ^3^ Due to the birds’ late age, few roosters were adequate for semen collection by abdominal massage, leading to a reduced sample size for the 63-week assay, and careful interpretation of results should be made.

**Table 7:** Composition of basal diets fed to breeder hens and roosters, with or without ZEN and anti-mycotoxin additive inclusion.

	Breeder Hen			Rooster
Age (Weeks)	22–25	26–40	41–65	22–65
Ingredients (%)				
Corn	61.93	58.29	48.84	57.16
Soybean	13.77	16.72	13.97	5.17
Wheat bran	19.77	13.75	23.04	33.57
Limestone	1.89	6.53	7.24	1.34
Soybean oil	0.00	1.85	4.29	0.00
Dicalcium phosphate	1.55	1.63	1.34	1.44
Vitamin and mineral premix ^1^	0.40	0.40	0.40	0.40
Salt	0.28	0.28	0.29	0.29
Sodium bicarbonate	0.20	0.20	0.20	0.20
DL-Methionine	0.12	0.12	0.12	0.11
L-Lysine-HCL	0.08	0.03	0.05	0.12
L-Threonine	0.01	0.00	0.00	0.00
Inert (Sand) ^2^	0.00	0.20	0.20	0.20
Total	100.00	100.00	100.00	100.00
Zearalenone (C + ZEN and C + AM + ZEN), ppb	0.00	1000	1000	500
Anti-mycotoxin feed additive (C + AM and C + AM + ZEN), kg/MT	0.00	2.00	2.00	1.00
Calculated Composition				
Metabolizable energy, kcal/kg	2.800	2.800	2.800	2.700
Calcium, %	1.20	3.00	3.20	0.95
Available phosphorus, %	0.42	0.42	0.38	0.42
Sodium, %	0.20	0.20	0.20	0.20
Clorum, %	0.21	0.21	0.21	0.22
Potassium, %	0.60	0.60	0.60	0.60
Linoleic acid, %	1.58	2.73	3.98	1.62
Crude protein, %	15.00	15.00	14.50	13.00
Dig arginine, %	0.88	0.88	0.86	0.71
Dig Isoleucine, %	0.53	0.53	0.48	0.38
Dig Lysine, %	0.63	0.63	0.60	0.50
Dig SAA, %	0.55	0.55	0.52	0.48
Dig Methionine, %	0.33	0.33	0.31	0.28
Dig Threonine, %	0.47	0.47	0.45	0.37
Did Triptofan, %	0.15	0.15	0.15	0.12
Dig Valine, %	0.59	0.59	0.56	0.48

^1^ Guaranteed levels per Kg of feed: Folic acid (minimum) 175.00 mg/kg. Pantothenic acid (minimum) 2500.00 mg/kg. Bacillus subtilis (minimum) 2.00 × 1011, UFC/kg. Biotin (minimum) 6.25 mg/kg. Copper (minimum) 2500.00 mg/kg. Coline (minimum) 84.63 g/kg. Iron (minimum) 12.50 g/kg. Phytase (minimum) 75,000.00 FTU/kg. Iodine (minimum) 375.00 mg/kg. Manganese (minimum) 25.00 g/kg. Methionine (minimum) 198.00 g/kg. Niacin (minimum) 7500.00 g/kg. Selenium (minimum) 75.00 mg/kg. Vitamin A (minimum) 3,000,000.00 U.I./kg. Vitamin B1 (minimum) 375.00 mg/kg. Vitamin B12 (minimum) 2500.00 mcg/kg. Vitamin B2 (minimum) 1500 mg/kg. Vitamin B6 (minimum) 500.00 mg/kg. Vitamin D3 (minimum) 750,000.00 U.I./kg. Vitamin E (minimum) 12,500.00 U.I./kg. Vitamin K3 (minimum) 800.00 mg/kg. Zinc (minimum) 25.00 mg/kg. Virginiamycin (minimum) 5000.00 mg/kg. ^2^ Soybean oil was partially replaced by sand to correct the energy content of the diets.

**Table 8:** Mycotoxin quantification in experimental diets.

Mycotoxins (μg/kg)	C ^1^	C + AM	C + ZEN	C + AM + ZEN
Aflatoxin B1	<QL ^2^	<QL	<QL	<QL
Aflatoxin B2	<QL	<QL	<QL	<QL
Aflatoxin G1	<QL	<QL	<QL	<QL
Aflatoxin G2	<QL	<QL	<QL	<QL
Fumonisin B1	679.5	159.9	572.4	179.4
Fumonisin B2	198.2	<QL	151.5	<QL
Zearalenone	<QL	<QL	846.6	1254.0
Deoxynivalenol	<QL	<QL	<QL	<QL
Ochratoxin	<QL	<QL	<QL	<QL
Toxin T2	<QL	<QL	<QL	<QL

^1^ Control (C)—standard corn and soybean-based diet; C + AM—control diet with the inclusion of anti-mycotoxin additive; C + ZEN—control diet experimentally contaminated with zearalenone; C + AM + ZEN—control diet experimentally contaminated with zearalenone and with inclusion of anti-mycotoxin feed additive. ^2^ QL: Minimum quantification limit.

## Data Availability

The original contributions presented in this study are included in the article. Further inquiries can be directed to the corresponding author.

## Abbreviations

The following abbreviations are used in this manuscript:

AM	Anti-mycotoxin feed additive
ZEN	Zearalenone
SEM	Standard error of the mean
C	Control group
ZEL	Zearalenol
